# Association between cardiorespiratory fitness and risk of incident venous thromboembolism: a prospective cohort study

**DOI:** 10.1016/j.rpth.2026.106898

**Published:** 2026-08-03

**Authors:** Haofeng Zhou, Ruisheng Zhang, Zhongxing Jiang, Ying Guo, Lan Guo, Haopeng Ke, Kaixuan Lin, Fang Wang

**Affiliations:** 1 Department of Cardiology, Beijing Hospital National, Center for Gerontology, National Clinical Research Center for Gerontology, The Key Laboratory of Geriatrics of National Health Commission, Institute of Geriatric Medicine Chinese Academy of Medical Sciences & Peking Union, Medical College, Beijing, China; 2 Department of Cardiology, Guangdong Provincial People’s Hospital, Guangdong Academy of Medical Sciences, Southern Medical University, Guangzhou, China; 3 Department of Computer Science and Engineering, Guangzhou Huali College, Guangzhou, China; 4 Department of Cardiology, Zhongshan Hospital of Traditional Chinese Medicine Affiliated to Guangzhou University of Traditional Chinese Medicine (Zhongshan Hospital of Traditional Chinese Medicine), Zhongshan, China; 5 Beijing Key Laboratory of Geriatrics-Based and AI-Driven Pharmaceutical and Medical Device Evaluation and Translation, Beijing, China

**Keywords:** biomarkers, cardiorespiratory fitness, cohort studies, venous thromboembolism

## Abstract

**Background:**

Venous thromboembolism (VTE) is a leading cardiovascular disease with substantial burden, yet the association between cardiorespiratory fitness (CRF) and VTE risk remains inconclusive.

**Objectives:**

This study is aimed to investigate the association of CRF with incident VTE, and quantify the mediating effects of circulating biomarkers.

**Methods:**

A total of 63,755 participants from the UK Biobank without prior VTE were included. CRF was estimated using a submaximal cycle ergometer test and classified into low (<20th percentile), moderate (20th-60th percentile), and high (>60th percentile) categories, standardized by sex and 10-year age groups. VTE was ascertained through linkage to national health records. Cox proportional hazards models adjusted for potential confounders were used to estimate hazard ratios (HRs) and 95% CIs. Polygenic risk scores for VTE were categorized into low, middle, and high tertiles. Mediation analyses were performed to assess the contribution of circulating biomarkers.

**Results:**

During a median follow-up of 12.5 years, 1744 incident VTE cases were documented. Each 1-metabolic equivalents increment in CRF was associated with an 8% lower VTE risk (HR 0.92, 95% CI 0.89-0.95). Compared with low CRF, high CRF was associated with a 27% lower risk (HR 0.73, 95% CI 0.63-0.84). The inverse association was consistently observed across all genetic risk strata. C-reactive protein, alkaline phosphatase, gamma-glutamyl transferase, cystatin C, and creatinine were estimated to account for 21.5% of this association.

**Conclusion:**

Higher CRF is associated with a lower VTE risk, regardless of genetic susceptibility. Inflammation, hepatobiliary enzymes, and renal function markers may account for a portion of this association.

## Essentials

• Association of CRF with VTE is unclear.
• This study included 63,755 UK Biobank participants to examine CRF and incident VTE.
• Higher CRF is associated with lower VTE risk, even among those with high genetic risk.
• Higher CRF reduces VTE risk partly by lowering inflammation and improving liver and kidney health.

## 1 Introduction

Venous thromboembolism (VTE), including deep vein thrombosis (DVT) and pulmonary embolism (PE), ranks as the third most common cause of cardiovascular disease worldwide, following myocardial infarction and stroke [1,2]. Although VTE is uncommon before the age of 40 years, with an annual incidence of approximately 1 per 10,000, its rate increases markedly after age 45 years [[3], [4], [5]]. VTE also imposes a considerable economic burden, with annual healthcare cost estimated at €1.5 to €3.3 billion in Europe and US $7 to $10 billion in the United States [6,7]. Therefore, identifying and managing modifiable risk factors for VTE are of great clinical significance.

Cardiorespiratory fitness (CRF) reflects the integrated capacity of the circulatory, respiratory, and muscular systems to transport and utilize oxygen during sustained physical activity [8]. CRF tends to decline gradually after the age of 30 and serves as a strong predictor of metabolic disorders, cardiovascular disease, and all-cause mortality [[9], [10], [11]]. Although several prior studies have explored the association between CRF and VTE, their findings are inconsistent and constrained by notable limitations, such as narrow study populations, small sample sizes, and the use of nonexercise predictive models to estimate CRF [[12], [13], [14]]. Furthermore, genetic predisposition plays a significant role in VTE risk [15]. In other diseases, such as dementia, higher CRF has been shown to be associated with lower disease risk across different levels of genetic predisposition [16]. Nevertheless, it remains unclear whether a similar association exists for VTE among individuals with a high genetic risk. Higher CRF is associated with favorable physiological profiles, including lower levels of systemic inflammation, improved liver function, and better renal function, and these pathways may in turn influence VTE risk [[17], [18], [19], [20]]. However, the potential mediating role of these biomarkers in the association between CRF and VTE has not been investigated.

To address these knowledge gaps, this study aimed to investigate the association of CRF with incident VTE, and to examine whether higher CRF is associated with lower VTE risk even among individuals with high genetic predisposition. Additionally, we performed exploratory mediation analyses to assess the potential mediating roles of circulating biomarkers.

## 2 Methods

### 2.1 Study design and population

This study utilized data from the UK Biobank, a large-scale prospective cohort that recruited over 500,000 participants aged 37 to 73 years between March 2006 and October 2010 across 22 assessment centers in the United Kingdom. At baseline, participants underwent touchscreen questionnaires, physical measurements, biological sampling, imaging, and genetic profiling. Data collection methods have been described in detail previously [21]. Incident disease and mortality were ascertained via linkage to national health records and periodic follow-up visits. This study was conducted in accordance with the principles of the Declaration of Helsinki. The UK Biobank was approved by the North West–Haydock Research Ethics Committee (16/NW/0274), and all participants provided written informed consent before enrollment. The data used in this study were obtained from the UK Biobank under application ID 144894.

Among the 502,650 participants recruited, we excluded 522 participants who withdrew, 15,220 with a history of VTE at baseline, and 423,153 without CRF data. Finally, 63,755 participants were included in the analysis (Supplementary Figure 1).

### 2.2 Measurements of CRF

CRF was estimated using a 6-minute submaximal cycle ergometer test without gas analysis. Prior to assessment, participants completed a risk-stratification questionnaire to determine their exercise risk category, and then an individualized testing protocol was assigned. Throughout the test, participants were instructed to maintain a cycling speed of 60 revolutions per minute, and heart rate was continuously monitored using a 4-lead electrocardiogram. CRF, expressed as maximal oxygen consumption (VO_2_max), was calculated using the validated prediction equation developed by Gonzalez et al. [22]. This equation exhibited strong validity relative to directly measured VO_2_max, with a Pearson correlation coefficient ranging from 0.68 to 0.74 and a minimal bias of less than 0.3 mL/min/kg. For analyses treating CRF as a continuous variable, values were converted into maximal metabolic equivalent of task (MET), where 1 MET = 3.5 mL/min/kg. For categorical analyses, CRF was classified into 3 categories within each sex and 10-year age group (eg, females aged 50–59) to account for age- and sex-related differences in fitness. Within each specific stratum, CRF values were categorized using percentile cutoffs: low CRF was defined as <20th percentile, moderate as 20th to 60th percentiles, and high as >60th percentile, consistent with existing CRF literature [23]. These stratum-specific categories were then merged across all sex-age groups to form a single categorical variable for the entire cohort.

### 2.3 Calculation of polygenic risk scores

The polygenic risk scores (PRS) for VTE was obtained from the Standard PRS (Category 301) provided by the UK Biobank [24]. These scores were generated using a Bayesian approach applied to meta-analyzed summary statistics from external genome-wide association studies data. The scores were calculated for all UK Biobank participants following standard genetic quality control, which excluded individuals with high heterozygosity or missingness, sex chromosome aneuploidy, mismatch between genetically inferred and self-reported sex, or excessive relatedness within the cohort [25]. The scores were subsequently subjected to a principal component–based ancestry centering step to approximately center the score distributions on zero across all ancestries. Within ancestry groups, the score distributions were standardized to have approximately unit variance, which ensures that the PRS values were comparable across different ancestral backgrounds. Of the 63,755 participants in the primary analytical sample, PRS data were available for 61,146 individuals, who were stratified into low, middle, and high genetic risk groups according to PRS tertiles.

### 2.4 Circulating biomarkers

Blood samples were collected from consenting participants at recruitment. Following standardized protocols, samples were processed to separate components and stored until subsequent analysis [26]. The following circulating biomarkers were selected as candidate mediators based on their established biological relevance to both CRF and VTE: inflammatory markers (white blood cell count and C-reactive protein [CRP]), liver function indicators (alanine aminotransferase, alkaline phosphatase [ALP], aspartate aminotransferase, and gamma-glutamyl transferase [GGT]), and renal function markers (cystatin C and creatinine).

### 2.5 Outcome ascertainment

The study outcome was incident VTE, including DVT and PE, which was identified by the well-validated “first occurrence” algorithm in the UK Biobank. This algorithm ascertains health outcomes using three-character International Classification of Diseases, 10th Revision (ICD-10) codes extracted from hospital inpatient records, death registries, primary care data, and self-reported medical conditions [27]. Incident VTE was defined based on ICD-10 codes I26, I80, I81, and I82, while PE was defined by code I26, and DVT was defined by codes I80, I81, and I82 [28]. Participants with concurrent PE and DVT were counted once for the overall VTE analysis (at the date of the first VTE event) but were included separately in both subtype-specific analyses. For each study participant, the follow-up period commenced at the baseline assessment and terminated at the earliest occurrence of the first incident VTE event, death, or the cutoff date of available follow-up data. At the time of analysis, hospital admission data were available until October 31, 2022 for England, August 31, 2022 for Scotland, and May 31, 2022 for Wales.

### 2.6 Covariates

The following baseline variables were treated as potential confounders. Demographic variables included age, sex, ethnicity (White and others), educational level (university or college degree and others), and the Townsend deprivation index (continuous), which was used to quantify socioeconomic status. Lifestyle factors covered smoking status (never, former, and current), daily alcohol intake (grams continuous), physical activity level (MET-minutes/week, continuous), and diet quality. Diet quality was assessed based on intake of fruit and vegetables, fish, processed and red meat, where high scores indicated healthy dietary (0, 1, 2, and 3 point) [29]. Body mass index (BMI) was calculated as weight in kilograms divided by height in meters squared. Physical activity level was measured by the International Physical Activity Questionnaire. Medical covariates included fracture history in the past 5 years, cancers, atrial fibrillation, heart failure, and autoimmune diseases, use of oral estrogen, antiplatelet agents, and anticoagulants. Detailed codes defining diseases and medications are summarized in Supplementary Table 1. Missing data for covariates (missing rate: 0.05%-20.09%, Supplementary Table 2) were handled using multiple imputation by chained equations with 5 imputed datasets. Estimates from the imputed datasets were pooled using Rubin’s rules.

### 2.7 Statistical analysis

Baseline characteristics were summarized by CRF category. Normal continuous variables were expressed as mean (SD), while nonnormal variables were expressed as median (IQR), and categorical variables were presented as frequencies (percentages).

Cox proportional hazards regression models were used to estimate the hazard ratios (HRs) and 95% CIs for the associations of CRF with incident VTE. Four models were constructed. Model 1 was unadjusted. Model 2 adjusted for age, sex, ethnicity, education, Townsend deprivation index. Model 3 additionally adjusted for smoking status, alcohol intake, diet score, physical activity, fracture history, cancers, atrial fibrillation, heart failure, autoimmune diseases, oral estrogen, antiplatelet agents, and anticoagulants. Model 4 further adjusted for BMI. The proportional hazards assumption was assessed using the Schoenfeld residuals method. To investigate the dose–response relationship, restricted cubic spline analysis with 3 knots (25th, 50th, and 75th percentiles) was performed.

To determine whether high CRF mitigates the risk of genetic susceptibility, we first assessed the association between CRF and the incidence of VTE across different genetic risk strata. A multiplicative interaction test was performed to assess effect modification by PRS tertiles and CRF categories. Then, we constructed joint association models by defining 9 combined groups of PRS tertiles and CRF categories, with the high PRS and low CRF group as the reference.

Mediation effects were estimated via the CMAverse package. For each candidate mediator, 3 models were constructed with adjustment for all covariates included in the fully adjusted model: (1) linear regression models to estimate the association between CRF and the mediator; (2) Cox proportional hazards regression models to estimate the associations of CRF and the mediator with incident VTE; and (3) mediation models combining the linear and Cox regression frameworks to estimate the natural direct effect (NDE) and natural indirect effect. All effect estimates were expressed as HRs with 95% CIs. The proportion mediated was calculated as (total effect [TE] − NDE) / (TE − 1), where TE represents the total effect of CRF on VTE, with 95% CIs derived from 1000 nonparametric bootstrap resamples.

Subgroup analyses were conducted to evaluate potential effect modification by age (≤60 vs >60 years), sex, and BMI (<30 vs ≥30 kg/m^2^), physical activity level (<600 MET-min/week vs ≥600 MET-min/week), and cancer history. Interaction effects were tested by incorporating cross-product terms of the exposure variable and each stratification factor into the fully adjusted model. Sensitivity analyses were performed to verify the robustness of the results. First, we repeated the analysis after excluding cases diagnosed within the first 3 years of follow-up to minimize the potential reverse causation. Second, Fine and Gray proportional subdistribution hazards regression models were constructed to account for the potential competing risk of death. Third, we replaced BMI with waist circumference as a measure of obesity in the fully adjusted model.

All analyses were performed in the R version 4.4.3 (R Foundation for Statistical Computing). All statistical tests were two-tailed, and *P* < .05 was considered statistically significant.

## 3 Results

### 3.1 Baseline characteristics

A total of 63,755 participants were included in the analysis (mean age 56.5 years; 46.5% male). The baseline characteristics stratified by CRF category are presented in Table 1. Compared with those in the low CRF group, participants with high CRF were more likely to be younger, highly educated, less deprived. They also reported a healthier diet pattern, more physical activity, and a lower BMI. Participants with high CRF also exhibited a lower burden of comorbidities and less use of estrogen, antiplatelet agents, and anticoagulants.

**Table 1:** Baseline characteristics of participants.

Characteristic	Overall	Low CRF	Moderate CRF	High CRF
*N*	63755	12847	25399	25509
Age, y, mean (SD)	56.48 (8.1)	56.81 (8.2)	56.57 (8.2)	56.22 (8.1)
Male, *N* (%)	29640 (46.5)	5958 (46.4)	11787 (46.4)	11895 (46.6)
White ethnicity, *N* (%)	57669 (90.5)	11110 (86.5)	22837 (89.9)	23722 (93.0)
College or university degree, *N* (%)	23460 (36.8)	3671 (28.6)	8681 (34.2)	11108 (43.5)
Townsend deprivation index, median (IQR)	−1.8 (−3.4,0.8)	−1.4 (−3.2,1.5)	−1.8 (−3.5,0.7)	−1.9 (−3.6,0.6)
Smoking status, *N* (%)				
Never	35861 (56.2)	7271 (56.6)	14270 (56.2)	14320 (56.1)
Former	22045 (34.6)	4520 (35.2)	8735 (34.4)	8790 (34.5)
Current	5849 (9.2)	1056 (8.2)	2394 (9.4)	2399 (9.4)
Alcohol intake, g/day, median (IQR)	11.3 (2.3,23.7)	6.8 (1.1,21.4)	11.3 (2.3,23.7)	13.5 (4.2,24.8)
Diet score, *N* (%)				
0	10179 (16.0)	2439 (19.0)	4189 (16.5)	3551 (13.9)
1	23832 (37.4)	4930 (38.4)	9711 (38.2)	9191 (36.0)
2	21339 (33.5)	4024 (31.3)	8392 (33.0)	8923 (35.0)
3	8405 (13.2)	1454 (11.3)	3107 (12.2)	3844 (15.1)
Physical activity, MET-min/week, median (IQR)	1879 (895, 3684)	1485 (674, 3072)	1756.00 (825, 3527)	2213 (1130, 4104)
BMI, kg/m^2^, mean (SD)	27.1 (4.5)	31.3 (5.1)	27.5 (3.6)	24.5 (3.0)
History of fracture, *N* (%)	5547 (8.7)	1084 (8.4)	2165 (8.5)	2298 (9.0)
Cancers, *N* (%)	5354 (8.4)	1134 (8.8)	2210 (8.7)	2010 (7.9)
Atrial fibrillation, *N* (%)	806 (1.3)	295 (2.3)	286 (1.1)	225 (0.9)
Heart failure, *N* (%)	182 (0.3)	72 (0.6)	63 (0.2)	47 (0.2)
Autoimmune diseases, *N* (%)	784 (1.2)	214 (1.7)	327 (1.3)	243 (1.0)
Estrogen, *N* (%)	2453 (3.8)	399 (3.1)	1004 (4.0)	1050 (4.1)
Antiplatelet agents, *N* (%)	6789 (10.6)	1912 (14.9)	2832 (11.2)	2045 (8.0)
Anticoagulants, *N* (%)	327 (0.5)	166 (1.3)	86 (0.3)	75 (0.3)

BMI, body mass index; CRF, cardiorespiratory; MET, metabolic equivalent of task.

### 3.2 Associations of CRF with VTE

**Figure 1:**
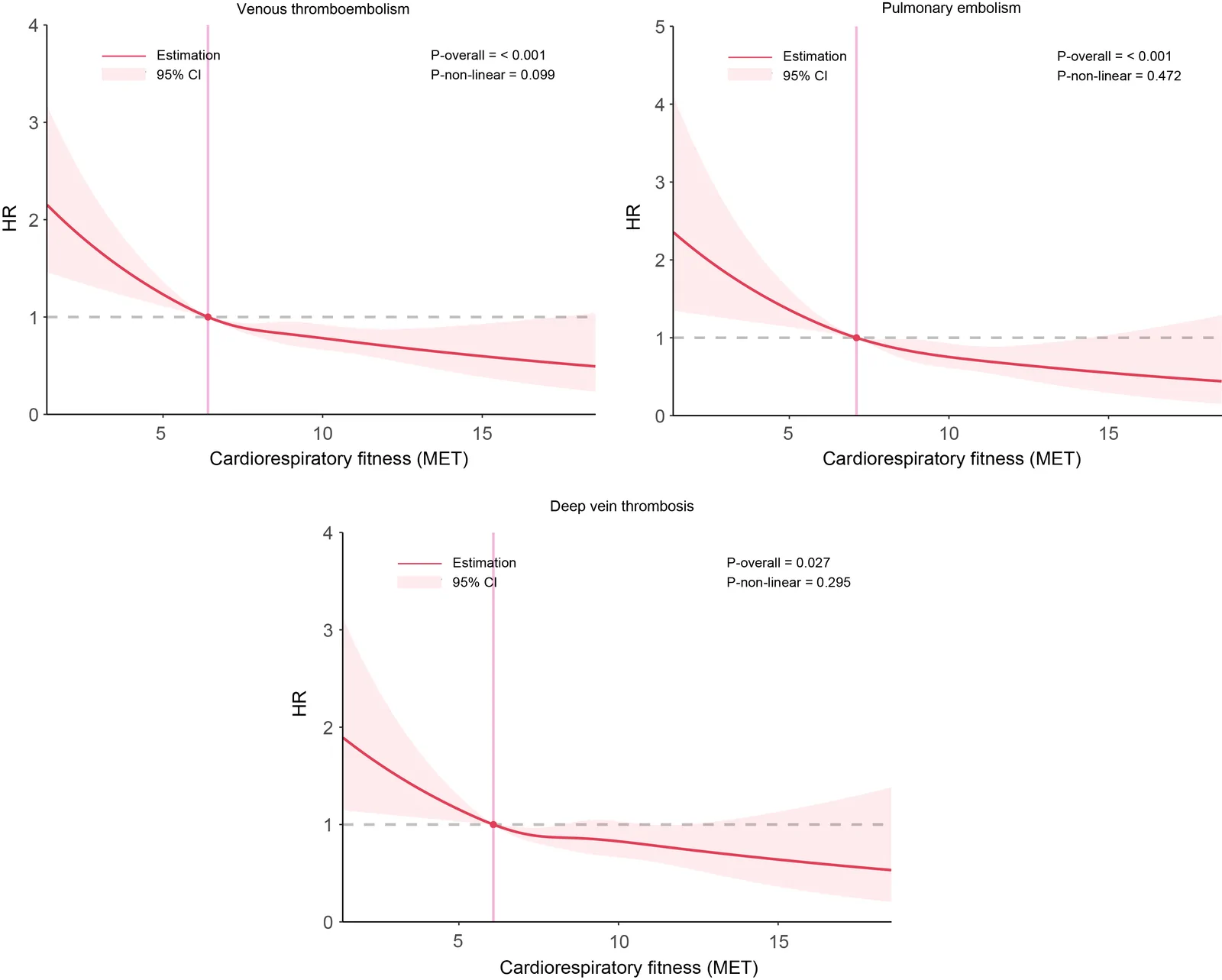
Restricted cubic spline curves for the associations of cardiorespiratory fitness with venous thromboembolism. HR, hazard ratio; MET, metabolic equivalent of task.

During a median follow-up period of 12.50 (IQR 12.40-12.64) years, 1744 incident VTE cases were identified, including 936 PE and 997 DVT. After full adjustment, higher CRF was significantly associated with a lower risk of incident VTE (Table 2). Per 1-MET increment in CRF was associated with an 8% reduced risk of VTE (HR 0.92, 95% CI 0.89-0.95). Compared with individuals in the low CRF group, those with moderate and high CRF had a 12% (HR 0.88, 95% CI 0.78-0.99) and 27% (HR 0.73, 95% CI 0.63-0.84) lower risk of VTE (*P* for trend < .001). Each 1-MET increment in CRF was associated with a 10% lower risk of PE (HR 0.90, 95% CI 0.85-0.94) and a 6% lower risk of DVT (HR 0.94, 95% CI 0.90-0.98). Participants with high CRF had a 34% lower risk of PE (HR 0.66, 95% CI 0.54-0.81) and a 20% lower risk of DVT (HR 0.80, 95% CI 0.66-0.97) compared with those in the low CRF group. Restricted cubic spline analysis revealed a linear association between CRF and the incidence of overall VTE, DVT, and PE (all *P* nonlinear > .05; Figure 1).

**Table 2:** Associations of cardiorespiratory fitness with venous thromboembolism.

Cardiorespiratory fitness	Events/person-years	Model 1		Model 2		Model 3		Model 4	
		HR (95%CI)	*P*	HR (95%CI)	*P*	HR (95%CI)	*P*	HR (95%CI)	*P*
**Venous thromboembolism**									
Per 1 MET increase	1744/789,621	0.91 (0.89-0.93)	<.001	0.88 (0.85-0.90)	<.001	0.89 (0.86-0.92)	<.001	0.92 (0.89-0.95)	<.001
Low	470/158,521	Reference		Reference		Reference		Reference	
Moderate	726/314,431	0.78 (0.69-0.87)	<.001	0.79 (0.71-0.89)	<.001	0.79 (0.70-0.88)	<.001	0.88 (0.78-0.99)	.027
High	548/316,669	0.58 (0.52-0.66)	<.001	0.60 (0.53-0.68)	<.001	0.60 (0.53-0.68)	<.001	0.73 (0.63-0.84)	<.001
*P* for trend			<.001		<.001		<.001		<.001
**Pulmonary embolism**									
Per 1 MET increase	936/794,482	0.89 (0.86-0.92)	<.001	0.86 (0.82-0.90)	<.001	0.85 (0.82-0.89)	<.001	0.90 (0.85-0.94)	<.001
Low	262/159,734	Reference		Reference				Reference	
Moderate	398/316,452	0.76 (0.65-0.89)	<.001	0.78 (0.67-0.92)	.003	0.77 (0.66-0.91)	.001	0.86 (0.73-1.02)	.086
High	276/318,296	0.53 (0.45-0.63)	<.001	0.57 (0.48-0.68)	<.001	0.55 (0.46-0.66)	<.001	0.66 (0.54-0.81)	<.001
*P* for trend			<.001		<.001		<.001		<.001
**Deep vein thrombosis**									
Per 1 MET increase	997/792,969	0.93 (0.90-0.96)	<.001	0.90 (0.86-0.94)	<.001	0.90 (0.86-0.93)	<.001	0.94 (0.90-0.98)	.012
Low	260/159,564	Reference		Reference				Reference	
Moderate	404/315,891	0.79 (0.68-0.93)	.003	0.80 (0.69-0.93)	.005	0.79 (0.68-0.93)	.004	0.90 (0.77-1.06)	.234
High	333/317,514	0.65 (0.55-0.76)	<.001	0.67 (0.57-0.79)	<.001	0.65 (0.55-0.77)	<.001	0.80 (0.66-0.97)	.022
*P* for trend			<.001		<.001		<.001		.021

HR, hazard ratio; MET, metabolic equivalent of task.

Model 1 was unadjusted. Model 2 adjusted for age, sex, ethnicity, education level, and Townsend deprivation index. Model 3 additionally adjusted for smoking status, alcohol intake, diet score, physical activity level, fracture history, cancers, atrial fibrillation, heart failure, autoimmune diseases, oral estrogen, antiplatelet agents, and anticoagulants. Model 4 further adjusted for body mass index.

### 3.3 Associations of CRF and genetic risk with VTE

**Figure 2:**
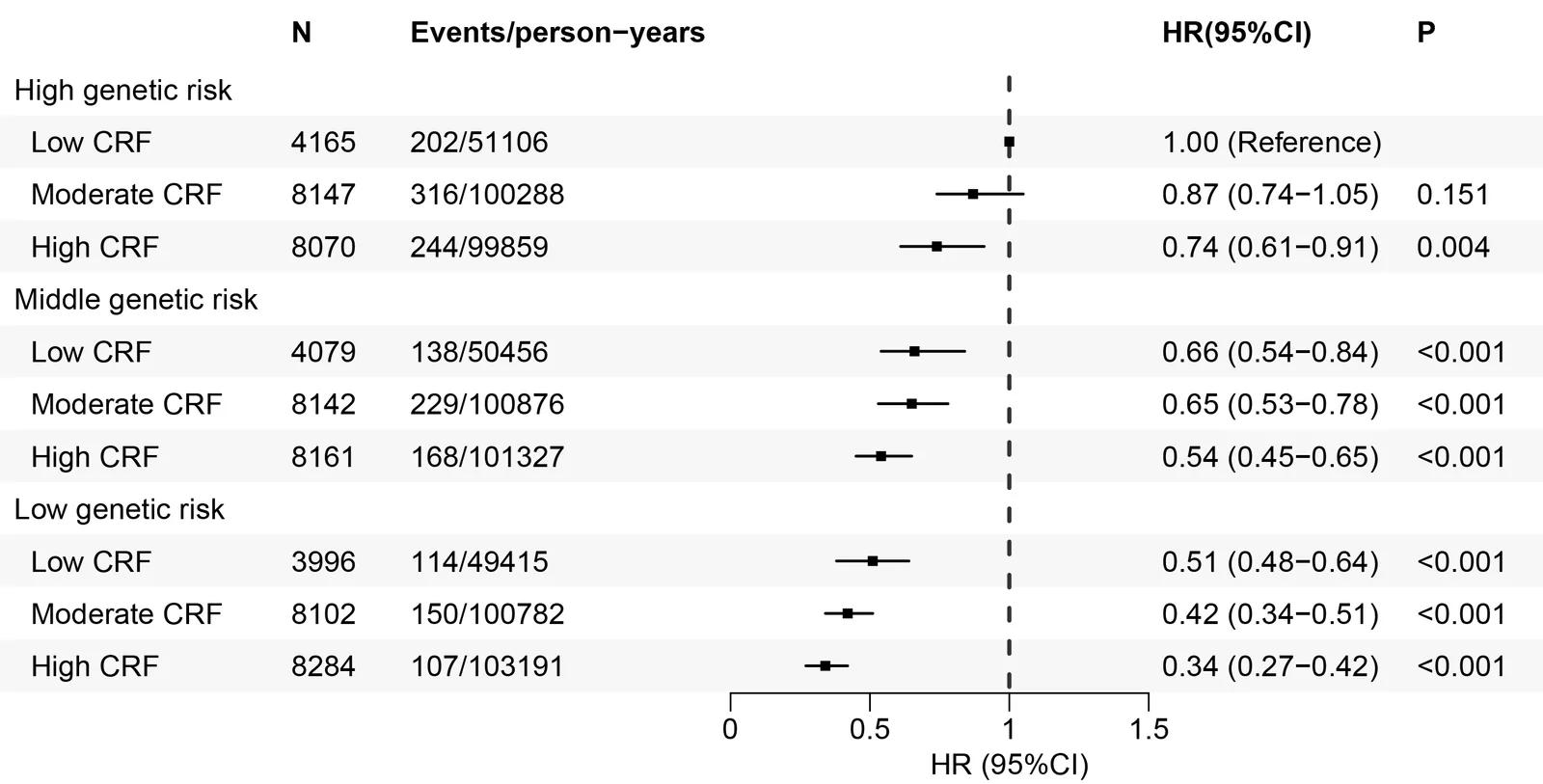
Joint associations of cardiorespiratory fitness and genetic risk with venous thromboembolism. CRF, cardiorespiratory fitness; HR, hazard ratio.

Compared with the low-risk group, both middle and high genetic risk were associated with a higher risk of VTE (middle: HR = 1.44, 95% CI: 1.26-1.65; high: HR = 2.06, 95% CI: 1.81-2.33; Supplementary Table 3). In stratified analyses using tertiles of the PRS, high CRF was associated with lower VTE risk across all genetic risk categories, and no significant interaction was observed between CRF categories and PRS tertiles (*P* for interaction = .336; Supplementary Table 4). In joint analysis using the high genetic risk and low CRF group as the reference, participants with high CRF in the high genetic risk group still exhibited a 26% lower risk (HR 0.74, 95% CI 0.61-0.91); notably, those with low genetic risk and high CRF had the lowest VTE risk (HR 0.34, 95% CI 0.27-0.42; Figure 2).

### 3.4 Mediation analysis

In mediation analyses, CRP, ALP, GGT, cystatin C, and creatinine significantly mediated the association between CRF and VTE risk, with individual mediation proportions of 3.5% for CRP, 6.6% for ALP, 6.4% for GGT, 7.6% for cystatin C, and 0.7% for creatinine. Collectively, these 5 mediators accounted for 21.5% of the total association between CRF and VTE (Table 3). For PE and DVT subtypes, mediation was primarily driven by ALP, GGT, cystatin C, and creatinine, with total mediation proportions of 15.4% and 24.2%, respectively (Supplementary Tables 5 and 6).

**Table 3:** Association of cardiorespiratory fitness with incident venous thromboembolism mediated by blood biomarkers.

Biomarkers	Total effect		Natural direct effect		Natural indirect effect		Proportion mediated		*P*
	HR	95% CI	HR	95% CI	HR	95% CI	%	95% CI	
White blood cell count	0.922	0.888-0.952	0.922	0.885-0.951	1.000	0.996-1.010	0.2	−7% to 4%	.884
C-reactive protein	0.922	0.891-0.958	0.924	0.893-0.962	0.997	0.994-1.000	3.5	2.7%-9.3%	<.001
Alanine aminotransferase	0.921	0.886-0.945	0.919	0.882-0.941	1.001	0.998-1.010	−2.9	−9.7% to 0.5%	.101
Aspartate aminotransferase	0.922	0.888-0.963	0.922	0.887-0.965	1.000	0.998-1.001	0.5	−0.5% to 1.6%	.312
Alkaline phosphatase	0.922	0.892-0.952	0.927	0.897-0.958	0.994	0.993-0.996	6.6	3.7%-11.9%	<.001
Gamma-glutamyl transferase	0.923	0.892-0.956	0.928	0.897-0.959	0.995	0.993-0.997	6.4	3.5%-11.5%	<.001
Cystatin C	0.923	0.886-0.962	0.929	0.892-0.968	0.994	0.992-0.995	7.6	5.0%-17.0%	<.001
Creatinine	0.921	0.887-0.955	0.922	0.888-0.955	0.999	0.998-1.000	0.7	0.3%-1.9%	<.001
Total							21.5	13.1%-38.5%	<.001

HR, hazard ratio.

Models adjusted for age, sex, ethnicity, education level, Townsend deprivation index, smoking status, alcohol intake, diet score, physical activity level, body mass index, fracture history, cancers, atrial fibrillation, heart failure, autoimmune diseases, oral estrogen, antiplatelet agents, and anticoagulant.

### 3.5 Subgroup and sensitivity analyses

The association between CRF and VTE risk was consistently observed in strata defined by age, sex, BMI, physical activity level, smoking status, and cancer history, with no significant interactions detected (all *P* for interaction > .05; Supplementary Table 7). In sensitivity analyses, after excluding 266 cases occurring within the first 3 years of follow-up, the results remained consistent. In Fine–Gray competing risk models accounting for 4064 competing deaths, the association between CRF and VTE risk was not materially altered (Supplementary Table 8). Replacing BMI with waist circumference in the fully adjusted model yielded results similar to those of the primary analysis.

## 4 Discussion

In this large-scale prospective cohort study utilizing data from the UK Biobank, we found that higher CRF was associated with a lower risk of incident VTE. Notably, the observed association of high CRF with VTE risk was consistently observed across all strata of genetic risk, including those with high genetic susceptibility. Furthermore, mediation analysis suggested systemic inflammation, hepatobiliary enzymes, and renal function markers were estimated to account for approximately one-fifth of the association between CRF and VTE.

This study extends the existing evidence regarding the association between CRF and VTE. Zöller et al. [12] first reported that higher weight-adjusted maximal workload was associated with a 19% lower risk of VTE in adulthood, but their sample comprised only men, and VTE events occurred predominantly before age 50 years, limiting its generalizability to older adults and women. However, Kunutsor et al. [13] reported no significant association between CRF and VTE risk among 2249 middle-aged men. Evensen et al. [14] observed an inverse association using an estimated CRF from a nonexercise model in a general population, yet its reliance on a nonexercise prediction model for CRF may introduce measurement bias. This study employed a validated submaximal cycle ergometer test to estimate CRF, which balances feasibility and objectivity. In this large, population-based sample with long follow-up, we provide robust evidence that higher CRF is significantly associated with lower VTE risk.

Genetic factors are a well-established determinant of VTE risk, with individuals at high genetic risk exhibiting a twofold higher incidence of VTE in this cohort. Prior research showed that high CRF is associated with lower risk of other diseases, such as coronary heart disease, across different levels of genetic predisposition [30]. To our knowledge, this is the first study to demonstrate that the inverse association between high CRF and lower VTE risk is consistently observed even among individuals in the highest tertile of genetic risk. In this subgroup, those with high CRF exhibited a 26% lower risk compared with their unfit counterparts. This finding indicates that lifestyle factors which improve CRF could be associated with lower incidence of VTE even in the context of high genetic predisposition. Furthermore, the lowest VTE risk was observed in participants with both low genetic risk and high CRF, highlighting the potential of considering both genetic and lifestyle factors in VTE risk assessment.

The underlying mechanisms linking CRF to VTE risk involve multiple pathways. From a hemodynamic perspective, higher CRF reflects enhanced cardiac efficiency and improved venous return, thereby reducing venous stasis [31,32]. Additionally, higher CRF is associated with better skeletal muscle function, particularly in the lower limbs, where an effective calf muscle pump facilitates venous blood return and reduces pooling [33,34]. The mediation analyses further reveal biological pathways through which CRF reduces VTE risk. First, systemic inflammation appeared to partially mediate this association. Elevated CRP induces monocytes to express tissue factor, impair endogenous fibrinolysis, and activate the coagulation cascade, thereby increasing thrombotic risk [35,36]. The antiinflammatory effects of CRF was well established [31]. Regular physical activity, a key determinant of CRF, can reduce the expression of proinflammatory proteins and, therefore, reduce thrombotic risk [37]. Second, hepatobiliary enzymes, including ALP and GGT, mediated larger proportions of the association. Elevated liver enzymes may reflect liver injury and are associated with increased VTE risk, potentially through oxidative stress and dysregulation of coagulation factors [[38], [39], [40]]. Third, renal function markers, particularly cystatin C, also significantly mediated the association. Cystatin C, a marker of kidney function, may capture shared pathways involving endothelial dysfunction and coagulation abnormalities [41,42]. Although creatinine also showed a statistically significant mediation effect, its contribution was quantitatively minimal, which might support cystatin C as a more sensitive biomarker in this pathway.

Our findings have important clinical implications. First, our results advocate for the incorporation of CRF assessment into clinical practice for VTE risk stratification. Second, the linear relationship between CRF and VTE risk suggests that any improvement in fitness could translate into a measurable reduction in population-level VTE burden [43]. The strengths of this study include the large sample size, prospective design, objectively measured CRF, and integration of genetic risk data. However, several limitations should be acknowledged. First, CRF assessment was completed by only a subset of UK Biobank participants. Individuals retained for analysis were healthier and of higher socioeconomic status compared with those excluded (Supplementary Table 9), and this selection bias likely attenuates the observed association toward the null. Second, VTE diagnosis was retrieved through the ICD-10, which may cause misclassification. Third, despite adjustment for numerous covariates, residual confounding from unmeasured factors may persist, such as D-dimer and fibrinogen. Fourth, CRF and covariates assessment were performed at baseline, and there was a significant time gap between the baseline assessment and the study end point, which could introduce some bias as these variables may change. Fifth, the UK Biobank population is predominantly of European ancestry, limiting the generalizability to other ethnic groups.

## 5 Conclusion

In this large prospective cohort study, higher CRF was significantly associated with a lower risk of incident VTE in a linear manner, and this inverse association was consistently observed across all strata of genetic risk. Mediation analyses suggested inflammation, hepatobiliary enzymes, and renal function markers as mediators, collectively explaining approximately one-fifth of the total association. These findings suggest that CRF may be a modifiable risk factor for VTE and support the value of integrating CRF assessment and promotion into VTE prevention strategies.

## References

[1] Di Nisio M., van Es N., Büller H.R. (2016). Deep vein thrombosis and pulmonary embolism. Lancet.

[2] Martin S.S., Aday A.W., Almarzooq Z.I., Anderson C.A.M., Arora P., Avery C.L. (2024). 2024 Heart disease and stroke statistics: a report of US and global data from the American Heart Association. Circulation.

[3] Cushman M. (2007). Epidemiology and risk factors for venous thrombosis. Semin Hematol.

[4] Wendelboe A.M., Raskob G.E. (2016). Global burden of thrombosis: epidemiologic aspects. Circ Res.

[5] Law Y., Chan Y.C., Cheng S.W.K. (2018). Epidemiological updates of venous thromboembolism in a Chinese population. Asian J Surg.

[6] Barco S., Woersching A.L., Spyropoulos A.C., Piovella F., Mahan C.E. (2016). European Union-28: an annualised cost-of-illness model for venous thromboembolism. Thromb Haemost.

[7] Grosse S.D., Nelson R.E., Nyarko K.A., Richardson L.C., Raskob G.E. (2016). The economic burden of incident venous thromboembolism in the United States: a review of estimated attributable healthcare costs. Thromb Res.

[8] Ross R., Blair S.N., Arena R., Church T.S., Després J.-P., Franklin B.A. (2016). Importance of assessing cardiorespiratory fitness in clinical practice: a case for fitness as a clinical vital sign: a scientific statement from the American Heart Association. Circulation.

[9] Zhou H., Wang Y., Song X., Xu T., Xia C., Guo Y. (2026). Independent and joint association of fat-to-muscle mass ratio and cardiorespiratory fitness with type 2 diabetes mellitus incidence: a prospective cohort study. Diabetes Obes Metab.

[10] Kokkinos P., Faselis C., Samuel I.B.H., Pittaras A., Doumas M., Murphy R. (2022). Cardiorespiratory fitness and mortality risk across the spectra of age, race, and sex. J Am Coll Cardiol.

[11] Chen Y., Yang H., Li D., Zhou L., Lin J., Yin X. (2025). Association of cardiorespiratory fitness with the incidence and progression trajectory of cardiometabolic multimorbidity. Br J Sports Med.

[12] Zöller B., Ohlsson H., Sundquist J., Sundquist K. (2017). Cardiovascular fitness in young males and risk of unprovoked venous thromboembolism in adulthood. Ann Med.

[13] Kunutsor S.K., Mäkikallio T.H., Araújo C.G.S., Jae S.Y., Kurl S., Laukkanen J.A. (2019). Cardiorespiratory fitness is not associated with risk of venous thromboembolism: a cohort study. Scand Cardiovasc J.

[14] Evensen L.H., Isaksen T., Braekkan S.K., Hansen J.B. (2019). Cardiorespiratory fitness and future risk of venous thromboembolism. J Thromb Haemost.

[15] Lutsey P.L., Zakai N.A. (2023). Epidemiology and prevention of venous thromboembolism. Nat Rev Cardiol.

[16] Wang S., Xu L., Yang W., Wang J., Dove A., Qi X. (2025). Association of cardiorespiratory fitness with dementia risk across different levels of genetic predisposition: a large community-based longitudinal study. Br J Sports Med.

[17] Bouglé D., Zunquin G., Sesbouë B., Sabatier J.P. (2010). Relationships of cardiorespiratory fitness with metabolic risk factors, inflammation, and liver transaminases in overweight youths. Int J Pediatr.

[18] Paluch A.E., Pool L.R., Isakova T., Lewis C.E., Mehta R., Schreiner P.J. (2019). Association of fitness with racial differences in chronic kidney disease. Am J Prev Med.

[19] Rayes J., Brill A. (2024). Hot under the clot: venous thrombogenesis is an inflammatory process. Blood.

[20] Ribic C., Crowther M. (2016). Thrombosis and anticoagulation in the setting of renal or liver disease. Hematology Am Soc Hematol Educ Program.

[21] Sudlow C., Gallacher J., Allen N., Beral V., Burton P., Danesh J. (2015). UK biobank: an open access resource for identifying the causes of a wide range of complex diseases of middle and old age. PLoS Med.

[22] Gonzales T.I., Westgate K., Strain T., Hollidge S., Jeon J., Christensen D.L. (2021). Cardiorespiratory fitness assessment using risk-stratified exercise testing and dose-response relationships with disease outcomes. Sci Rep.

[23] Kaze A.D., Santhanam P., Ahima R.S., Bertoni A.G., Echouffo-Tcheugui J.B. (2024). Association between microvascular disease and cardiorespiratory fitness among adults with type 2 diabetes. Diabetes Care.

[24] Thompson D.J., Wells D., Selzam S., Peneva I., Moore R., Sharp K. (2024). A systematic evaluation of the performance and properties of the UK Biobank Polygenic Risk Score (PRS) Release. PLoS One.

[25] Bycroft C., Freeman C., Petkova D., Band G., Elliott L.T., Sharp K. (2018). The UK Biobank resource with deep phenotyping and genomic data. Nature.

[26] Elliott P., Peakman T.C., Biobank U.K. (2008). The UK Biobank sample handling and storage protocol for the collection, processing and archiving of human blood and urine. Int J Epidemiol.

[27] Xu J., Ren Q., Su Y., Lin L., Shen R., Huang K. (2025). Frailty, genetic risk, and long-term risk of venous thromboembolism: insight from a UK Biobank study. Am J Hematol.

[28] Xiang H., Gan X., Zhang Y., Zhang Y., Ye Z., Yang S. (2025). Association of social isolation and plasma metabolites with the risk of venous thromboembolism. Arterioscler Thromb Vasc Biol.

[29] Li Z., Cheng S., Guo B., Ding L., Liang Y., Shen Y. (2025). Wearable device-measured moderate to vigorous physical activity and risk of degenerative aortic valve stenosis. Eur Heart J.

[30] Collings P.J., Wang M., Chen Z., Shi Q., Luo S., Yeung S.L.A. (2026). Cardiorespiratory fitness, genetic risk, and incident atherosclerotic cardiovascular events: a prospective cohort study. Mayo Clin Proc.

[31] Lavie C.J., Arena R., Swift D.L., Johannsen N.M., Sui X., Lee D.-C. (2015). Exercise and the cardiovascular system: clinical science and cardiovascular outcomes. Circ Res.

[32] Rowland T.W. (2001). The circulatory response to exercise: role of the peripheral pump. Int J Sports Med.

[33] Mau T., Lui L.Y., Distefano G., Kramer P.A., Ramos S.V., Toledo F.G.S. (2023). Mitochondrial energetics in skeletal muscle are associated with leg power and cardiorespiratory fitness in the study of muscle, mobility and aging. J Gerontol A Biol Sci Med Sci.

[34] Houghton D.E., Ashrani A., Liedl D., Mehta R.A., Hodge D.O., Rooke T. (2021). Reduced calf muscle pump function is a risk factor for venous thromboembolism: a population-based cohort study. Blood.

[35] Ding J., Yue X., Tian X., Liao Z., Meng R., Zou M. (2023). Association between inflammatory biomarkers and venous thromboembolism: a systematic review and meta-analysis. Thromb J.

[36] Bisoendial R.J., Kastelein J.J.P., Levels J.H.M., Zwaginga J.J., van den Bogaard B., Reitsma P.H. (2005). Activation of inflammation and coagulation after infusion of C-reactive protein in humans. Circ Res.

[37] Li Q.Q., Qin K.R., Zhang W., Guan X.M., Cheng M., Wang Y.X. (2023). Advancements in the regulation of different-intensity exercise interventions on arterial endothelial function. Rev Cardiovasc Med.

[38] Motaganahalli R.L., Kapoor R., Timsina L.R., Gutwein A.R., Ingram M.D., Raman S. (2021). Clinical and laboratory characteristics of patients with novel coronavirus disease-2019 infection and deep venous thrombosis. J Vasc Surg Venous Lymphat Disord.

[39] Lee D.H., Jacobs D.R. (2005). Association between serum gamma-glutamyltransferase and C-reactive protein. Atherosclerosis.

[40] Folsom A.R., Lutsey P.L., Roetker N.S., Rosamond W.D., Lazo M., Heckbert S.R. (2014). Elevated hepatic enzymes and incidence of venous thromboembolism: a prospective study. Ann Epidemiol.

[41] Cheung K.L., Zakai N.A., Callas P.W., Howard G., Mahmoodi B.K., Peralta C.A. (2018). Mechanisms and mitigating factors for venous thromboembolism in chronic kidney disease: the REGARDS study. J Thromb Haemost.

[42] Demirkol S., Cakar M., Balta S., Unlu M., Kurt O., Akhan M. (2013). Serum cystatin C levels should correlate with endothelial dysfunction and inflammation indirectly through renal function. Arq Bras Cardiol.

[43] Myers J., Kokkinos P., Arena R., LaMonte M.J. (2021). The impact of moving more, physical activity, and cardiorespiratory fitness: why we should strive to measure and improve fitness. Prog Cardiovasc Dis.

