# Supplementary material for: Association between cardiorespiratory fitness and risk of incident venous thromboembolism: a prospective cohort study
Source: Res Pract Thromb Haemost. 2026 Aug 3;10(5):106898. doi: 10.1016/j.rpth.2026.106898 (PMC13508637; doi:10.1016/j.rpth.2026.106898)
Supplement: Supplementary file 1 [file mmc1.docx]

Figure S1. Flowchart of the selection of the study population

VTE, venous thromboembolism; CRF, cardiorespiratory fitness.

Table S1. Field ID and code defining diseases and medications in this study

|  | Filed ID | Code |
| --- | --- | --- |
| Venous thromboembolism | 131308, 131309, 131396, 131397, 131400, 131401 | I26, I80, I81, I82 |
| Pulmonary embolism | 131308, 131309 | I26 |
| Deep vein thrombosis | 131396, 131397, 131400, 131401 | I80, I81, I82 |
| Fracture in the past 5 years | 2463 |  |
| Cancers | 2453 |  |
| Atrial fibrillation | 131350, 131351 | I48 |
| Heart failure | 131354, 131355 | I50 |
| Autoimmune diseases | 131626-131629, 131848-131851, 131890-131897 | K50, K51, M05, M06, M30-33 |
| Oral estrogen | 6153 |  |
| Antiplatelet agents | 20003 | 1140868226, 1140861806, 1141168318, 1141168322, 1140861778, 1140861780, 1141167848, 1141167844 |
| Anticoagulants | 20003 | 1140888266, 1140910832, 1140864956, 1140881842, 1140888204, 1140888206, 1140926444, 1141171364, 1141189054, 1140861506, 1140861588, 1140861604 |

Table S2. Information on missing variables in analysis

|  | Number | Percentage |
| --- | --- | --- |
| Ethnicity | 36 | 0.06% |
| Townsend deprivation index | 280 | 0.44% |
| Educational level | 747 | 1.17% |
| Smoking status | 362 | 0.57% |
| Alcohol intake | 58 | 0.09% |
| Physical activity | 12809 | 20.09% |
| Diet score | 6867 | 10.81% |
| Body mass index | 35 | 0.05% |

Table S3. Associations of genetic risk with incident venous thromboembolism

| Genetic risk group | Events/person-years | Model 1 | | Model 2 | |
| --- | --- | --- | --- | --- | --- |
|  |  | HR (95%CI) | *P* | HR (95%CI) | *P* |
| Low genetic risk | 371/253388 | Reference |  | Reference |  |
| Middle genetic risk | 535/252659 | 1.45 (1.27-1.65) | <0.001 | 1.44 (1.26-1.65) | <0.001 |
| High genetic risk | 762/251252 | 2.06 (1.82-2.32) | <0.001 | 2.06 (1.81-2.33) | <0.001 |

Model 1 was unadjusted. Molde 2 adjusted for age, sex, ethnicity, education level, Townsend deprivation index, smoking status, alcohol intake, diet score, physical activity level, body mass index, fracture history, cancers, atrial fibrillation, heart failure, autoimmune diseases, oral estrogen, antiplatelet agents and anticoagulants.

Table S4. Stratified analysis of cardiorespiratory fitness with incident venous thromboembolism within each genetic risk category

| Risk group | Low CRF | Moderate CRF | |  | High CRF | |  | P for interaction |
| --- | --- | --- | --- | --- | --- | --- | --- | --- |
|  |  | HR (95% CI) | *P* |  | HR (95% CI) | *P* |  |  |
| Low genetic risk | Reference | 0.75 (0.58-0.97) | 0.028 |  | 0.57 (0.44-0.82) | <0.001 |  | 0.336 |
| Middle genetic risk | Reference | 0.88 (0.69-1.16) | 0.329 |  | 0.79 (0.62-0.98) | 0.037 |  |  |
| High genetic risk | Reference | 0.86 (0.71-1.04) | 0.128 |  | 0.73 (0.59-0.90) | 0.002 |  |  |

Models adjusted for age, sex, ethnicity, education level, Townsend deprivation index, smoking status, alcohol intake, diet score, physical activity level, body mass index, fracture history, cancers, atrial fibrillation, heart failure, autoimmune diseases, oral estrogen, antiplatelet agents and anticoagulants.

Table S5. Association of cardiorespiratory fitness with incident pulmonary embolism mediated by blood biomarkers

| Biomarkers | Total effect | |  | Natural direct effect | |  | Natural indirect effect | |  | Proportion mediated | |  |
| --- | --- | --- | --- | --- | --- | --- | --- | --- | --- | --- | --- | --- |
|  | HR | 95% CI |  | HR | 95% CI |  | HR | 95% CI |  | % | 95% CI | P |
| White blood cell count | 0.897 | 0.848-0.944 |  | 0.897 | 0.846-0.945 |  | 1.000 | 0.995-1.007 |  | 0.3% | -7.4%-4.2% | 0.758 |
| C-reactive protein | 0.897 | 0.852-0.948 |  | 0.899 | 0.853-0.948 |  | 0.988 | 0.996-1.003 |  | 1.8% | -1.6%-4.2% | 0.261 |
| Alanine aminotransferase | 0.897 | 0.847-0.933 |  | 0.893 | 0.844-0.927 |  | 1.001 | 1.000-1.010 |  | -3.3% | -12.9%-0.2% | 0.083 |
| Aspartate aminotransferase | 0.897 | 0.854-0.951 |  | 0.898 | 0.853-0.952 |  | 1.000 | 0.999-1.000 |  | 0.1% | -10.6%-1.1% | 0.906 |
| Alkaline phosphatase | 0.897 | 0.858-0.943 |  | 0.901 | 0.864-0.947 |  | 0.995 | 0.993-0.999 |  | 4.2% | 0.4%-8.9% | 0.041 |
| Gamma-glutamyl transferase | 0.899 | 0.858-0.933 |  | 0.905 | 0.863-0.940 |  | 0.994 | 0.992-0.997 |  | 5.4% | 2.7%-9.9% | 0.020 |
| Cystatin C | 0.898 | 0.862-0.937 |  | 0.904 | 0.868-0.943 |  | 0.993 | 0.991-0.995 |  | 5.8% | 3.7%-11.7% | <0.001 |
| Creatinine | 0.896 | 0.854-0.933 |  | 0.897 | 0.854-0.934 |  | 0.999 | 0.999-1.000 |  | 0.5% | 0.08%-1.1% | 0.022 |
| Total |  |  |  |  |  |  |  |  |  | 15.4% | 8.5%-38.9% | <0.001 |

Models adjusted for age, sex, ethnicity, education level, Townsend deprivation index, smoking status, alcohol intake, diet score, physical activity level, body mass index, fracture history, cancers, atrial fibrillation, heart failure, autoimmune diseases, oral estrogen, antiplatelet agents and anticoagulants.

Table S6. Association of cardiorespiratory fitness with incident deep venous thrombosis mediated by blood biomarkers

| Biomarkers | Total effect | |  | Natural direct effect | |  | Natural indirect effect | |  | Proportion mediated | |  |
| --- | --- | --- | --- | --- | --- | --- | --- | --- | --- | --- | --- | --- |
|  | HR | 95% CI |  | HR | 95% CI |  | HR | 95% CI |  | % | 95% CI | P |
| White blood cell count | 0.943 | 0.894-0.981 |  | 0.942 | 0.891-0.980 |  | 1.001 | 0.997-1.012 |  | -1.2% | -2.5%-7.7% | 0.923 |
| C-reactive protein | 0.943 | 0.888-0.985 |  | 0.946 | 0.890-0.986 |  | 0.997 | 0.994-1.002 |  | 4.7% | -2.9%-19.2% | 0.212 |
| Alanine aminotransferase | 0.943 | 0.905-0.991 |  | 0.942 | 0.903-0.989 |  | 1.003 | 0.998-1.012 |  | -1.8% | -22.9%-3.6% | 0.537 |
| Aspartate aminotransferase | 0.943 | 0.903-0.991 |  | 0.944 | 0.904-0.992 |  | 0.999 | 0.999-1.001 |  | 1.2% | -0.1%-8.7% | 0.084 |
| Alkaline phosphatase | 0.943 | 0.904-0.977 |  | 0.949 | 0.909-0.984 |  | 0.994 | 0.992-0.996 |  | 8.1% | 3.2%-29.5% | 0.008 |
| Gamma-glutamyl transferase | 0.944 | 0.903-0.991 |  | 0.948 | 0.907-0.995 |  | 0.996 | 0.994-0.999 |  | 6.8% | 0.4%-36.2% | 0.037 |
| Cystatin C | 0.944 | 0.902-0.990 |  | 0.950 | 0.906-0.995 |  | 0.994 | 0.992-0.996 |  | 9.9% | 4.7%-36.3% | <0.001 |
| Creatinine | 0.943 | 0.898-0.990 |  | 0.943 | 0.898-0.989 |  | 0.999 | 0.999-1.000 |  | 0.8% | 0.02%-4.7% | 0.045 |
| Total |  |  |  |  |  |  |  |  |  | 24.2% | 17.2%-48.1% | 0.018 |

Models adjusted for age, sex, ethnicity, education level, Townsend deprivation index, smoking status, alcohol intake, diet score, physical activity level, body mass index, fracture history, cancers, atrial fibrillation, heart failure, autoimmune diseases, oral estrogen, antiplatelet agents and anticoagulants.

Table S7. Subgroup analyses for associations of cardiorespiratory fitness with incident venous thromboembolism

| Subgroup | Low CRF | Moderate CRF | |  | High CRF | |  | *P* for interaction |
| --- | --- | --- | --- | --- | --- | --- | --- | --- |
|  |  | HR (95% CI) | *P* |  | HR (95% CI) | *P* |  |  |
| Age |  |  |  |  |  |  |  | 0.307 |
| < 60 years | Reference | 0.82 (0.69-1.00) | 0.041 |  | 0.68 (0.55-0.86) | 0.001 |  |  |
| ≥ 60 years | Reference | 0.95 (0.81-1.12) | 0.561 |  | 0.79 (0.65-0.96) | 0.016 |  |  |
| Sex |  |  |  |  |  |  |  | 0.067 |
| Female | Reference | 0.78 (0.66-0.94) | 0.008 |  | 0.70 (0.57-0.86) | 0.001 |  |  |
| Male | Reference | 0.98 (0.83-1.15) | 0.781 |  | 0.75 (0.62-0.92) | 0.005 |  |  |
| BMI category |  |  |  |  |  |  |  | 0.687 |
| < 30 kg/m^2^ | Reference | 0.93 (0.79-1.12) | 0.436 |  | 0.76 (0.63-0.91) | 0.003 |  |  |
| ≥ 30 kg/m^2^ | Reference | 0.86 (0.72-1.02) | 0.091 |  | 0.78 (0.57-1.09) | 0.158 |  |  |
| Physical activity |  |  |  |  |  |  |  | 0.795 |
| < 600 MET-min/week | Reference | 0.91 (0.69-1.22) | 0.495 |  | 0.81 (0.57-1.15) | 0.251 |  |  |
| ≥ 600 MET-min/week | Reference | 0.88 (0.78-1.01) | 0.07 |  | 0.73 (0.62-0.85) | <0.001 |  |  |
| History of cancer |  |  |  |  |  |  |  | 0.134 |
| Yes | Reference | 1.16 (0.82-1.66) | 0.338 |  | 0.87 (0.58-1.32) | 0.529 |  |  |
| No | Reference | 0.85 (0.75-0.98) | 0.014 |  | 0.71 (0.61-0.83) | <0.001 |  |  |

Models adjusted for age, sex, ethnicity, education level, Townsend deprivation index, smoking status, alcohol intake, diet score, physical activity level, body mass index, fracture history, cancers, atrial fibrillation, heart failure, autoimmune diseases, oral estrogen, antiplatelet agents and anticoagulants.

Table S8. Sensitivity analyses for associations of cardiorespiratory fitness with incident venous thromboembolism

|  | Accounting for competing risk of death | | Excluding cases that occurred within the first 3 years | |  | Replacing BMI with waist circumference | |
| --- | --- | --- | --- | --- | --- | --- | --- |
|  | HR (95%CI) | *P* | subdistribution HR (95%CI) | *P* |  | HR (95%CI) | *P* |
| Venous thromboembolism | |  |  |  |  |  |  |
| Per 1 MET increase | 0.92 (0.88-0.96) | <0.001 | 0.93 (0.90-0.97) | <0.001 |  | 0.91 (0.88-0.94) | <0.001 |
| Low CRF | Reference |  | Reference |  |  | Reference |  |
| Moderate CRF | 0.89 (0.78-1.02) | 0.096 | 0.90 (0.79-1.03) | 0.112 |  | 0.82 (0.73-0.92) | <0.001 |
| High CRF | 0.73 (0.63-0.86) | <0.001 | 0.76 (0.66-0.89) | <0.001 |  | 0.68 (0.59-0.78) | <0.001 |
| P for trend |  | <0.001 |  | <0.001 |  |  | <0.001 |
| Pulmonary embolism | |  |  |  |  |  |  |
| Per 1 MET increase | 0.89 (0.85-0.94) | <0.001 | 0.91 (0.87-0.96) | <0.001 |  | 0.89 (0.84-0.93) | <0.001 |
| Low CRF | Reference |  | Reference | 0.001 |  | Reference |  |
| Moderate CRF | 0.88 (0.74-1.05) | 0.177 | 0.87 (0.74-1.05) | 0.142 |  | 0.81 (0.69-0.95) | 0.009 |
| High CRF | 0.68 (0.55-0.84) | <0.001 | 0.72 (0.58-0.88) | 0.001 |  | 0.64 (0.53-0.77) | <0.001 |
| P for trend |  | <0.001 |  | 0.007 |  |  | <0.001 |
| Deep venous thrombosis | |  |  |  |  |  |  |
| Per 1 MET increase | 0.93 (0.89-0.99) | 0.016 | 0.95 (0.90-1.00) | 0.046 |  | 0.92 (0.88-0.97) | 0.001 |
| Low CRF | Reference |  | Reference |  |  | Reference |  |
| Moderate CRF | 0.89 (0.75-1.08) | 0.241 | 0.93 (0.78-1.10) | 0.291 |  | 0.82 (0.71-0.97) | 0.018 |
| High CRF | 0.77 (0.63-0.96) | 0.018 | 0.79 (0.62-0.99) | 0.028 |  | 0.73 (0.61-0.87) | 0.001 |
| P for trend |  | 0.027 |  | 0.041 |  |  | 0.009 |

BMI, body mass index.

Models adjusted for age, sex, ethnicity, education level, Townsend deprivation index, smoking status, alcohol intake, diet score, physical activity level, body mass index, fracture history, cancers, atrial fibrillation, heart failure, autoimmune diseases, oral estrogen, antiplatelet agents and anticoagulants.

Table S9. Baseline characteristics of participants included in and excluded from the analysis

| Characteristic | Included in analysis |  | Excluded from analysis |
| --- | --- | --- | --- |
| N | 63755 |  | 423153 |
| Age, years, mean (SD) | 56.48 (8.13) |  | 56.44 (8.10) |
| Male, N (%) | 29640 (46.5) |  | 193160 (45.6) |
| White ethnicity, N (%) | 57669 (90.5) |  | 399548 (94.4) |
| College or university degree, N (%) | 23182 (36.8) |  | 134192 (32.4) |
| Townsend deprivation index, median (IQR) | -1.79 (-3.44, 0.83) |  | -2.19 (-3.68, 0.50) |
| Smoking status, N (%) |  |  |  |
| Never | 35622 (56.2) |  | 230138 (54.7) |
| Former | 21962 (34.6) |  | 145206 (34.5) |
| Current | 5809 (9.2) |  | 45313 (10.8) |
| Alcohol intake, g/day, median (IQR) | 11.27 (2.25, 23.67) |  | 12.10 (0.00, 24.80) |
| Diet score, N (%) |  |  |  |
| 0 | 9058 (15.9) |  | 60520 (16.1) |
| 1 | 21279 (37.4) |  | 141574 (37.7) |
| 2 | 19016 (33.4) |  | 125015 (33.3) |
| 3 | 7535 (13.2) |  | 48824 (13.0) |
| Physical activity, MET-min/week, median (IQR) | 1866 (888, 3666) |  | 1777 (810, 3546) |
| BMI, kg/m^2^, mean (SD) | 27.09 (4.48) |  | 27.42 (4.80) |
| History of fracture, N (%) | 5539 (8.7) |  | 39804 (9.4) |
| Cancers, N (%) | 5354 (8.4) |  | 39043 (9.2) |
| Atrial fibrillation, N (%) | 806 (1.3) |  | 7035 (1.7) |
| Heart failure, N (%) | 182 (0.3) |  | 2268 (0.5) |
| Autoimmune diseases, N (%) | 784 (1.2) |  | 7173 (1.7) |
| Estrogen, N (%) | 2453 (3.8) |  | 16726 (4.0) |
| Antiplatelet agents, N (%) | 6789 (10.6) |  | 61098 (14.4) |
| Anticoagulants, N (%) | 327 (0.5) |  | 3291 (0.8) |

MET, metabolic equivalent of task; BMI, body mass index
